# A global comprehensive vaccine-preventable disease surveillance strategy for the immunization Agenda 2030^☆^

**DOI:** 10.1016/j.vaccine.2022.07.024

**Published:** 2024-04-08

**Authors:** Minal K. Patel, Heather M. Scobie, Fatima Serhan, Benjamin Dahl, Christopher S. Murrill, Tomoka Nakamura, Sarah W. Pallas, Adam L. Cohen, the Global Comprehensive Vaccine-Preventable Disease Surveillance Working Group

**Affiliations:** a Department of Immunization, Vaccines and Biologicals, World Health Organization, 20 Avenue Appia, 1211 Geneva, Switzerland; b Global Immunization Division, U.S. Centers for Disease Control and Prevention, 1600 Clifton Road, Atlanta, GA, USA

**Keywords:** Immunization, Surveillance, Vaccine-preventable disease

## Abstract

As part of the Immunization Agenda 2030, a global strategy for comprehensive vaccine-preventable disease (VPD) surveillance was developed. The strategy provides guidance on the establishment of high-quality surveillance systems that are 1) comprehensive, encompassing all VPD threats faced by a country, in all geographic areas and populations, using all laboratory and other methodologies required for timely and reliable disease detection; 2) integrated, wherever possible, taking advantage of shared infrastructure for specific components of surveillance such as data management and laboratory systems; 3) inclusive of all relevant data needed to guide immunization program management actions. Such surveillance systems should generate data useful to strengthen national immunization programs, inform vaccine introduction decision-making, and reinforce timely and effective detection and response. All stakeholders in countries and globally should work to achieve this vision.

## 1 Introduction

**Fig. 1:**
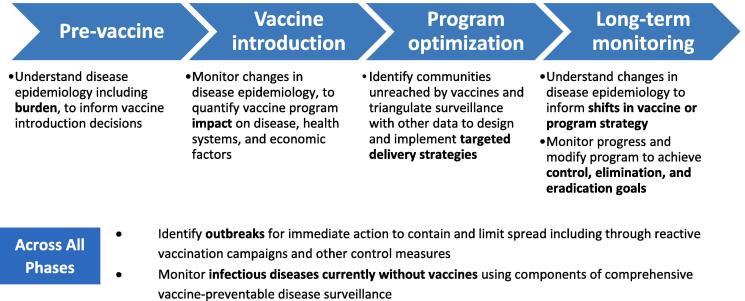
Why do countries conduct vaccine-preventable disease surveillance? Adapted from Cohen, A. *et al*. [15].

Surveillance for vaccine-preventable diseases (VPDs) provides critical data for timely detection and response to VPDs to guide optimal use of vaccines and other disease control measures, saving lives and lowering societal costs. VPD surveillance can help at various stages, from determining the disease burden and epidemiology for vaccine introductions, to updating or revising immunization schedules, to routine use of the data to identify under-served populations and achieve more equitable vaccine coverage, to evaluating vaccine impact and progress towards elimination or the need to optimize vaccination strategies (Fig. 1). Long-term surveillance data provides the evidence governments need on the impact of vaccines to prioritize and fund essential immunization programs (see Box 1).

Box 1 **Panel**

• The “Global Strategy on Comprehensive Vaccine-Preventable Disease Surveillance” extends the high-level global immunization strategy, *Immunization Agenda 2030 (IA2030): A Global Strategy to Leave No One Behind.* It summarizes a strategy for VPD surveillance for the period 2021–2030 and is of relevance to the following IA2030 Strategic Priorities:
• Strategic Priority 1: Immunization Programs for Primary Health Care / Universal Health Coverage.
• Key area of focus: Vaccine-preventable disease surveillance.
• Strategic Priority 5: Outbreaks & Emergencies.
• Key area of focus: Integrated surveillance.

Surveillance for VPDs forms part of wider infectious and non-infectious disease public health surveillance, i.e., the continuous and systematic collection, analysis and interpretation of health-related data needed for the planning, implementation, and evaluation of public health practice [1]. Surveillance systems, including those for VPDs, have a critical role to play in detecting and triggering responses to emerging and re-emerging infections, such as SARS-CoV-2. As such, surveillance is a core capacity that countries should develop, maintain and improve under the International Health Regulations (IHR), and it is an integral component of mechanisms to secure national and global health security. Over time, the need for high-quality surveillance data will continue to grow as progress is made towards elimination goals, as new diseases become vaccine-preventable, and as new and improved laboratory tests to confirm VPD cases are developed.

As part of the Immunization Agenda 2030, a technical annex entitled “Global Strategy on Comprehensive Vaccine-Preventable Disease Surveillance” was developed to outline the specific aims that the immunization community at national, regional, and global levels seeks to achieve with regards to VPD surveillance by 2030 [2].

Comprehensive VPD surveillance is defined as the country, regional and global systems that meet the World Health Organization (WHO)-recommended standards for surveillance of priority VPDs (as defined by each country), with integration of surveillance functions across VPDs and other diseases wherever possible [3]. The word “**comprehensive**” is used to indicate that surveillance for all priority VPDs, whatever form surveillance takes, should be considered an integral part of a country’s overall surveillance and national immunization strategy. This may require more robust implementation and potentially include additional VPDs, expanding laboratory networks, and expanding to geographic areas not currently included in national or sentinel VPD surveillance systems. Within a comprehensive VPD surveillance strategy, emphasis is placed on laboratory confirmation of the pathogen, epidemiological investigation, collection and reporting of individual-level (i.e., case-based) data, data management and analysis, and the visualization and use of VPD surveillance data for routine program monitoring, evaluation, optimization, decision-making and response.

The critical role VPD surveillance plays in public health is widely recognized, but until now there has been no global strategy to inform the national structure and integration of VPD surveillance in order to coordinate investments along the pathway to ever stronger, more effective, and resilient systems. Currently, most countries have national case-based VPD surveillance systems for polio, measles and neonatal tetanus; many countries also have sentinel case-based surveillance for one or more other VPDs [4]. In parallel, most countries have national notifiable disease reporting from health facilities, and some also have event-based surveillance to capture reports from the community and media for selected pathogens. However, current VPD surveillance in a given country is often fragmented, may not include all VPDs of importance to that country, and may not meet all the surveillance objectives in that specific country. Laboratory capacity for confirming and characterizing bacterial diseases and some viral diseases is limited in many countries. Furthermore, silos that exist between laboratories and epidemiologic units can result in the lack of linking of laboratory results to epidemiologic information for each case reported to the surveillance system, making the data challenging to interpret. Finally, some countries are challenged due to the lack of sustainable national funding for their surveillance systems. The global comprehensive VPD surveillance strategy provides a framework to bring together all types of surveillance for various VPDs encompassing viral and bacterial pathogens.

In many low-income countries, resources provided via the Global Polio Eradication Initiative (GPEI) support much of the VPD surveillance infrastructure even beyond polio surveillance [5]. There is a risk of losing that surveillance capacity and workforce as GPEI funding and other external donor funds to countries decline [6]. The global comprehensive VPD surveillance strategy suggests tailored approaches for building comprehensive VPD surveillance and identifies the need for external financing based on country capacity and income level, to complement countries’ increasing ownership of their surveillance systems funded increasingly from domestic resources over time as these systems mature [2].

Country-level VPD surveillance often does not meet the minimum recommended standards for many diseases, which limits the ability of countries and stakeholders to make evidence-based decisions [4]. The Strategic Advisory Group of Experts on Immunization (SAGE) has stated that poor data quality, including for VPD surveillance, was impeding program management and recommended that improving data quality should be a top priority for national immunization programs [7], [8]. In 2019, SAGE recommended strengthening governance and development of information systems; building capacity and capability of the health workforce for data generation and use; aligning information systems and technologic innovations with local context and program needs; and improving data sharing and use for continuous quality improvement [9], [10]. This strategy highlights the critical components of VPD surveillance needed to generate surveillance data to drive decision-making and policy.

## 2 The strategy

The purpose of a global strategy for comprehensive VPD surveillance is to address the current gaps and limitations of VPD surveillance in all countries. It does this by providing guiding principles for countries (1) to establish, maintain, and strengthen VPD surveillance, (2) to use surveillance data for public health action, and (3) to provide a monitoring and evaluation framework that countries and other stakeholders can use to assess the overall performance of VPD surveillance and to further invest in strengthening it.

The strategy was developed to achieve a vision where all countries have comprehensive, high-quality, sustainable VPD surveillance systems, supported by strong laboratory systems, that detect and confirm cases and outbreaks and generate usable information to guide outbreak prevention and response, immunization program optimization, and vaccination policymaking to decrease the burden of VPDs as efficiently, effectively, and equitably as possible. The aim is to secure and accelerate the development of country-led and -owned comprehensive VPD surveillance systems, including integration of support functions and resourcing across diseases. This strategy is intended to be used by all national and international immunization and surveillance stakeholders.

The strategy has five main objectives:

• To develop a **workforce** appropriately trained in surveillance core competencies, including data analysis and interpretation;
• To strengthen and expand public health **laboratory networks**;
• To develop sustainable, interoperable VPD surveillance **information systems** to support collection, analysis, sharing and programmatic use of data;
• To conduct **applied research** to enhance and monitor the quality of surveillance systems and their ability to adapt to new data needs, such as for new vaccines;
• To promote **sustainable financing** and increase domestic government support for core surveillance activities.

Comprehensive VPD surveillance should include, at a minimum, all VPDs with global surveillance mandates due to global elimination or eradication goals (currently polio, measles, and neonatal tetanus), IHR-targeted VPDs (smallpox, poliomyelitis due to wild-type poliovirus, and human influenza caused by a new subtype, and potentially notifiable VPDs including cholera, yellow fever, Ebola, and circulating vaccine-derived poliovirus), and other VPDs that are regional and country priorities [11], [12]. It should encompass the continued ability to conduct environmental surveillance of poliovirus and surveillance for antimicrobial resistance for relevant VPDs.

Comprehensive VPD surveillance should be able to provide:

• Routine reporting of surveillance data even in the absence of cases, i.e., zero case reporting, where applicable;
• Representative and complete detection of disease among the intended populations and geographic areas;
• Reliable laboratory confirmation of disease (where it is indicated);
• Efficient and timely collection of the relevant data needed for decision making, including:
  ◦ Case-based data on age and vaccination status, where indicated;
  ◦ Pinpointing of specific diseases by geographic location and affected groups to allow for risk assessment and evidence-based vaccine use;
  ◦ Identification of cases and outbreaks for epidemic-prone VPDs to support timely responses;
  ◦ Monitoring significant changes in disease epidemiology, including disease burden and the strains of pathogens, to guide vaccine development and use;
  ◦ Monitoring progress towards country, regional and global control, elimination, and eradication goals.

When deciding whether to conduct surveillance for a particular VPD, countries should consider whether surveillance will inform policy and immunization strategy decisions and whether resources and capacity are available. The specific scope and design of comprehensive VPD surveillance systems must be flexible and respond to a country’s needs. Because of that, it will differ across countries, encompassing different VPDs and using varying methodologies, including national case-based, sentinel case-based, aggregate notifiable disease, and event-based, according to specific surveillance objectives. Countries should decide which VPDs to include in their surveillance strategies based on national priorities [13].

A comprehensive VPD surveillance strategy builds on existing, high-quality surveillance systems, many of which already meet minimal standards and integrate some core or support functions. The intent of integration as part of this strategy is to provide a means to strengthen and broaden such systems rather than replace or duplicate them. Within a comprehensive approach, integration of specific surveillance functions can result in streamlined processes and deliver efficiencies across multiple diseases (including communicable diseases other than VPDs); these surveillance support functions can further serve to integrate VPD surveillance systems with each other and into other existing infectious and non-infectious disease surveillance systems (Table 1). Another example of integration is to adapt existing syndromic surveillance platforms to meet the surveillance standards for additional VPDs, as has been done for measles and rubella which have similar clinical presentations and can be tested using the same specimen.

**Table 1:** Potential areas of integration among surveillance support functions.

**Surveillance support functions**	**Potential areas for integration**
**Governance**	Standards and guidelines development, policy, laws/mandates, roles and responsibilities (including for private sector), funding
**Program management**	Budget creation, resource mobilization, financial management, sustainability, infrastructure/equipment management, human resources, external surveillance assessments and reviews
**Workforce capacity**	Training/capacity building at all levels; staff for core functions including case detection, notification, investigation, reporting, and response; epidemic preparedness
**Laboratory**	Specimen collection kits, reagents and supplies, equipment, physical space, and training; personnel; expansion and diversification of regional and global networks; shared procurement processes; quality management systems; laboratory information management systems
**Field logistics and communication**	Airtime and internet for notification and reporting, specimen collection and transport; feedback of results
**Supervision**	Supportive supervisory visits, workplans, checklists
**Data management and use**	Information system development; data harmonization, implementation, and use for performance improvement
**Coordination**	Linking surveillance program to relevant stakeholders (e.g., immunization program) for data review, dissemination and use; improvement planning; surveillance strengthening as core function of IHR implementation framework, including rapid response teams and Emergency Operations Centers

Development and implementation of comprehensive VPD surveillance systems should be monitored at the country, regional, and global levels. At country level, the national immunization program, the national disease control program in the Ministry of Health, and other governmental entities involved in VPD surveillance or response should be accountable for developing, implementing, monitoring, and financing a comprehensive VPD surveillance strategy, with input from national immunization technical advisory groups where relevant. At regional and global levels, WHO should be accountable for ensuring that countries have the necessary technical assistance to develop, implement, and monitor comprehensive VPD surveillance plans, with input and support from technical partners, including WHO Collaborating Centers and laboratories that are part of WHO networks.

## 3 Implementing the strategy

Implementing the comprehensive VPD surveillance strategy will require national commitment and strong coordinated action at country, regional, and global levels, including across existing structures and initiatives for specific VPDs (e.g., GPEI), broader infectious disease and event-based surveillance efforts (e.g., Integrated Disease Surveillance and Response [IDSR] in the Africa region), and public health system strengthening goals. To expand the scope of VPDs under surveillance to meet immunization program objectives, and to achieve the minimal recommended standards for each VPD included in countries’ prioritized set, will likely require additional resources beyond those currently committed. Estimation of the additional resource requirements to strengthen VPD surveillance over the 2021–2030 period has been undertaken in some regions (e.g., African region investment case for VPD surveillance [14]), and is underway at the global level. Preliminary estimates indicate that a minimum of US$3 billion in catalytic external funding would be required globally over the decade to supplement countries’ own investments in achieving comprehensive VPD surveillance, directed primarily to low-income and fragile countries (Supplemental Table 1; unpublished results). Such cost estimates will need to be updated regularly over the IA2030 period as different eradication, elimination, and control goals are achieved and as new diseases become vaccine-preventable and are included in countries’ surveillance systems.

## 4 Conclusions

History shows us the value of VPD surveillance. it has been critical for the eradication of smallpox, ongoing efforts to eradicate polio and eliminate measles and rubella, detecting and responding to VPD outbreaks, providing valuable data to guide evidence-based decision-making on new vaccine introductions, and documenting the full impact of vaccination programs on child mortality. However, VPD surveillance has not garnered the level of investment and advocacy that it needs to achieve what is expected of it. The COVID-19 pandemic has shown us the importance of high-quality disease surveillance, with the anticipation that COVID-19 will soon be classified as a VPD. There is an urgent need to maintain and strengthen VPD surveillance in all countries in a comprehensive and integrated way. This strategy provides a structure for countries and stakeholders to plan for and implement comprehensive VPD surveillance by training workforces, strengthening laboratories, better managing and using data, and ensuring sustainability.

## 5 Collaboration details

Working group: Jamal A. Ahmed, MD^3^, Nyambat Batmunkh, MD, MPH^4^, Myriam Ben Mamou, MD^5^, Claire Chauvin, MPH^3^, Danni Daniels^5^, Kamal Fahmy, PhD ^6^, Varja Grabovac, MSc^4^, Sudhir Joshi, MPH^7^, Balcha Masresha, MD^8^, Jason M. Mwenda, PhD^8^, Roberta Pastore, MD, MPH^4^, Gloria Rey-Benito^9^, Martha Velandia-Gonzalez ^9^

^3^Polio Eradication, World Health Organization, 20 Avenue Appia, 1211 Geneva, Switzerland

^4^World Health Organization Regional Office of the Western Pacific, P.O. Box 2932, 1000 Manila, Philippines

^5^World Health Organization Regional Office of Europe, UN City, Marmorvej 51, DK-2100 Copenhagen, Denmark

^6^World Health Organization Regional Office for the Eastern Mediterranean, Monazamet El Seha El Alamia Str, Extension of Abdel Razak El Sanhouri Street, P.O. Box 7608, Nasr City, Cairo 11371, Egypt

^7^World Health Organization Regional Office for South-East Asia, World Health House, Indraprastha Estate, Mahatma Gandhi Marg, New Delhi 110 002, India

^8^World Health Organization Regional Office for Africa, Cité du Djoué, P.O.Box 06 Brazzaville

Republic of Congo

^9^Pan American Health Organization, 525 23rd Street NW, Washington, DC 20037

## Declaration of Competing Interest

The authors declare that they have no known competing financial interests or personal relationships that could have appeared to influence the work reported in this paper.
