# Supplementary material for: A global comprehensive vaccine-preventable disease surveillance strategy for the immunization Agenda 2030
Source: Vaccine. 2024 Apr 8;42(Suppl 1):S124–8. doi: 10.1016/j.vaccine.2022.07.024 (PMC10746290; doi:10.1016/j.vaccine.2022.07.024)
Supplement: Supplementary data 1 [file mmc1.docx]

**Supplemental Table 1. Summary of approach for preliminary minimum estimate of external funding requirements for Comprehensive VPD Surveillance global strategy implementation, 2021-2030**

| **Dimension** | **Description** |
| --- | --- |
| Levels of costs included | - Global, regional, and country level activities |
| Countries included | - All low- and middle-income countries, as categorized by the World Bank in 2018 - Countries categorized based on country maturity model from Global Strategy on Comprehensive VPD Surveillance with adjustments for WHO Regional Office feedback and current country risk status:   - Category 1: Low-income countries + fragile states + polio endemic + high-risk polio countries + polio transition   - Category 1B: Lower- or upper-middle-income countries that are medium-high or regional high-risk polio countries + India   - Category 2: Lower-middle-income countries + Venezuela   - Category 3: Upper-middle-income countries + Kiribati |
| Funding sources included | - External donor funding to implementing partners, not including country government domestic resources or external donor in-kind contributions (e.g., donor staff costs) |
| Degree of external funding projected | - Largest degree of external funding for countries in Category 1 and least for countries in Category 3 |
| VPDs included^1^ | - Category 1 countries: Polio + Measles + Rubella + Neonatal Tetanus + Invasive Bacterial Disease (IBD)^2^ + (depending on region: Yellow Fever, Meningococcus, and/or Japanese Encephalitis) - Category 1b countries: Category 1 VPDs + Diphtheria + Rotavirus + (Typhoid or Pertussis) - Category 2 countries: Category 1 VPDs + Diphtheria + Rotavirus + (at least one of: Typhoid, Pertussis, and/or congenital rubella syndrome (CRS)) - Category 3 countries: Category 1 VPDs + Diphtheria + Rotavirus + Pertussis + (Typhoid and/or CRS) |
| VPD surveillance standard used | - Minimal recommended standards from WHO^3^ for all VPDs in a country tier’s prioritized set - Country performance against standards assessed based on Joint Reporting Form (JRF) and surveillance data submitted to WHO^4^ |
| Polio surveillance funding assumptions | - 2021-23: assumed GPEI Financial Resource Requirements (FRR) for surveillance (budget as of 2019), plus non-FRR contributions for surveillance and surveillance-related proportion of technical assistance and quality improvement (2018-19 expenditure levels plus inflation) - 2024-30: assumed polio Post-Certification Strategy financial estimate adjusted for inflation (with transition of acute flaccid paralysis surveillance from active (2024-26) to sentinel site (2027-28) to passive (2029-30) and with expansion of community-based, environmental, and primary immunodeficiency disease surveillance) |
| Non-polio VPD surveillance funding assumptions | - 2021-30: assumed level funding of current external donor commitments identified via Measles & Rubella Initiative, Gavi, U.S. CDC, and Korea CDC (adjusted for inflation, population growth for measles and rubella test kits, and including only recurrent costs of yellow fever test kits plus one-time equipment replacement) to maintain current surveillance breadth and quality for non-polio VPDs. - 2021-30: for each country that had not yet met the WHO-recommended minimal surveillance standard for the VPDs in its tier’s prioritized set, catalytic incremental funding was added for start-up costs (e.g., training, equipment purchase), recurrent costs (e.g., laboratory reagents, shipping, case investigation travel and per diem), and technical assistance and quality assurance costs (e.g., regional-level technical assistance missions, annual External Quality Assurance materials and shipping). Incremental funding packages were scaled by country category: start-up costs for categories 1-2; recurrent costs for categories 1-1b (and 2 for measles and rubella); technical assistance and quality assurance for categories 1-3. Start dates for incremental funding packages were staggered by region and year across the decade to reflect a programmatically realistic path to capacity building for new VPDs and improved quality surveillance. Data sources for incremental funding packages included subject matter experts from WHO and U.S. CDC, published and unpublished cost studies, UNICEF supply catalogue, project budgets, and other sources used in the external funding requirements estimate for internal consistency (e.g., salary scales, per diem rates). |
| Aggregate surveillance funding / integration assumptions | - Assumed existence of foundational government public health surveillance system in every country - Assumed minimal system integration (e.g., external funding for supplemental human resources, equipment, field logistics for specific VPDs would also support capacity for aggregate surveillance) |

(1) Adjustments made for some individual countries based on Comprehensive VPD Surveillance Working Group feedback and information about current VPDs under surveillance by country. (2) Invasive Bacterial Disease (IBD) sentinel site surveillance for *Haemophilus influenzae* and pneumococcus, and meningococcus in some cases, was included as a surrogate for capacity for any bacterial VPD surveillance because of greater availability of cost data; costs are expected to be comparable to other bacterial sentinel disease surveillance systems. (3) <https://www.who.int/immunization/monitoring_surveillance/burden/vpd/standards/en/> (4) <https://www.who.int/immunization/monitoring_surveillance/burden/vpd/JRF_Supplementary_Questionnaire_Surveillance_18Mar.pdf?ua=1>; <https://www.who.int/immunization/monitoring_surveillance/burden/VPDs/en/>
